# Non-traumatic Spinal Subdural Hemorrhage Associated With Rivaroxaban Use

**DOI:** 10.7759/cureus.59208

**Published:** 2024-04-28

**Authors:** Gabriel Niccolo P Navata, Pia Teresa A Camara

**Affiliations:** 1 Department of Clinical Neurosciences, University of the East Ramon Magsaysay Memorial Medical Center, Quezon City, PHL; 2 Department of Adult Neurology, Center for Neurological Sciences, Quirino Memorial Medical Center, Quezon City, PHL

**Keywords:** complete cord syndrome, non-valvular atrial fibrillation, rivaroxaban, non-traumatic spinal subdural hemorrhage, spontaneous spinal subdural hemorrhage

## Abstract

Spinal subdural hematoma (SSDH) is a rare medical emergency that can cause permanent neurological deficits. The disease is characterized by sudden onset back pain, sensorimotor changes, and bladder and autonomic dysfunction. This is often associated with the use of anticoagulants, blood dyscrasias, and recent spinal procedures. We present a case of a 63-year-old male maintained on rivaroxaban for nonvalvular atrial fibrillation clinically presenting with abrupt onset back pain that rapidly progressed to sensorimotor deficits and bladder dysfunction. Rivaroxaban, a selective inhibitor of factor Xa, has been approved by the Food and Drug Administration (FDA) for the reduction of stroke risk and systemic embolism in nonvalvular atrial fibrillation. We postulate that rivaroxaban played a major role in triggering the spinal hemorrhage. This case highlights the very limited documented cases of spontaneous subdural spinal hemorrhages associated with rivaroxaban use.

## Introduction

Spinal subdural hematoma (SSDH) is a rare condition regarded as a medical emergency as it can lead to rapidly progressive neurological deficits [1]. It is often characterized by sudden onset of back pain or headache followed by sensorimotor changes as well as bladder and autonomic dysfunction [2,3]. The most common causative factors are anticoagulant therapy, dysfunction of blood coagulation, and iatrogenic spinal procedures [3]. Spontaneous SSDH is observed in the absence of any associated trauma or iatrogenic causes (i.e., spinal tap, insertion of epidural catheters, or spinal surgeries) [1].

The rate of mortality for spontaneous nontraumatic SSDH has been decreasing in recent years, and it is currently estimated at 1.3%. However, morbidity, including permanent neurological deficits, is significantly higher at approximately 28% [4]. While many hypotheses have been proposed, the pathogenesis of spontaneous SSDH still remains poorly understood [1].

To the best of our knowledge, there are very few documented accounts of spontaneous SSDH associated with rivaroxaban therapy. In this article, we report a case of a 63-year-old male maintained on rivaroxaban who presented with SSDH confirmed by MRI of the spine.

## Case presentation

A 63-year-old male, with a history of hypertension and permanent atrial fibrillation, maintained on rivaroxaban 15 mg once daily, was gardening when he presented with sudden onset vague lower back pain while standing. No immediate medical evaluation was done, and the patient opted to sit down and rest instead. However, an hour later, the lower back pain gradually became more severe and was now associated with an inability to move and feel both lower extremities, as well as an inability to urinate. This prompted medical consultation at the emergency department. The patient had no prior history of trauma or spinal procedures.

On examination, the patient was awake and oriented with a Glasgow coma scale (GCS) score of 15. His vital signs included a blood pressure of 140/100 mmHg; a heart rate was irregular at 65 beats per minute; a respiratory rate of 18 cycles per minute, and a body temperature of 36.0°C. Back pain was graded 9/10 in intensity. The neurological evaluation showed a Medical Research Council (MRC) grading of 5/5 for motor strength in his bilateral upper extremities and 0/5 on all of the muscle groups of the bilateral lower extremities. A sensory examination showed a complete absence of sensation in his bilateral lower extremities at the level of the L2 dermatome. Muscle stretch reflexes were absent over the bilateral lower extremities. A bladder examination revealed a palpable distended bladder with no urge to urinate.

The initial blood chemistry revealed a platelet count of 161,000/uL, an elevated prothrombin time of 18.5 (range: 10-12) with an international normalized ratio of 1.54, and a percentage activity of 43.6%. Partial thromboplastin time was 35.4 (range: 29-34). Clotting time and bleeding time were measured at 10 (range: 8-15) and two minutes (range: 1-3), respectively. Other serum tests yielded normal results. An electrocardiogram confirmed the presence of atrial fibrillation.

**Figure 1:**
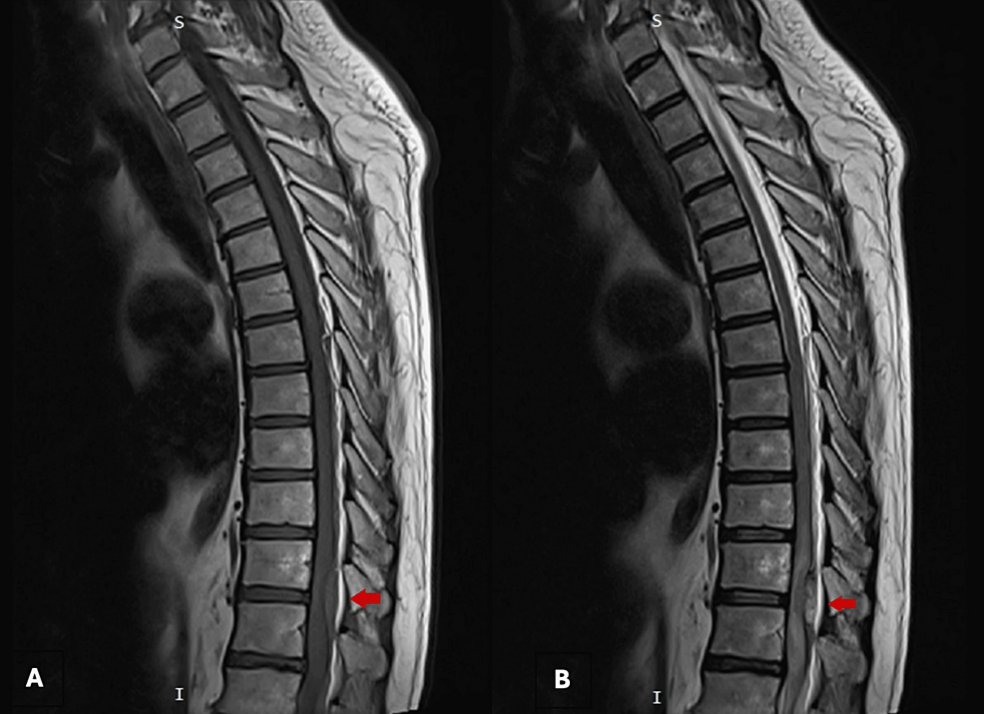
T1- and T2-weighted imaging of the spine. (A) T1-weighted sagittal image of the thoracic spine illustrates a homogeneous hyperintense crescent-shaped abnormality measuring 0.9 x 1.0 x 2.2 cm in anteroposterior, transverse, and craniocaudal dimensions, respectively. This fluid collection is observed compressing the dorsal aspect of the thecal sac and spinal cord at the T11 and T12 levels. (B) Counterpart T2-weighted imaging shows that this fluid collection is heterogeneous with consequent spinal cord compression and edema at the same level. These depict a spontaneous extra-axial hemorrhage that is subdural in location.

**Figure 2:**
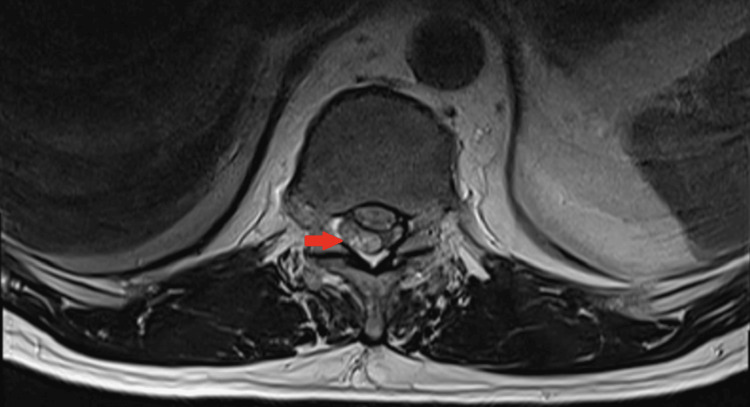
T2-weighted axial MRI at the level of the T12 illustrates the right-lateralized subdural heterogeneous fluid collection (red arrow).

An MRI of the thoracic and lumbosacral spine with and without contrast was performed. T1- and T2-weighted images revealed a crescent-shaped intradural, extramedullary heterogeneous signal at the level of T11 and T12 vertebrae with marked compression of the dorsal aspect of the spinal cord at this level, resulting in spinal cord edema (see Figures 1, 2).

The patient was admitted, and because of the presence of neurological impairment, the patient was advised to undergo immediate surgical intervention. However, he opted to refuse because of financial constraints. Anticoagulation was discontinued, and pain medication was started for the back pain. Examination throughout the entire hospital stay showed no improvement in the motor strengths of the bilateral lower extremities as well as sensation over the L2 dermatome and below.

After a week of hospitalization, the patient maintained refusal of neurosurgical intervention and opted to be discharged against medical advice. Upon discharge, the neurological evaluation showed no signs of improvement: 0/5 strength and a complete absence of sensation over the bilateral lower extremities. Moreover, he had no bowel or bladder control and was advised on self-catheterization techniques and the use of suppositories.

## Discussion

The last few years have seen an increase in the popularity and usage of direct oral anticoagulants (DOACs). Currently, rivaroxaban, dabigatran, apixaban, and edoxaban are the four available warfarin substitutes [5]. Rivaroxaban (Xarelto) acts as a selective inhibitor of factor Xa, which decreases prothrombinase activity and, consequently, thrombin generation [6]. Its Food and Drug Administration (FDA)-approved uses include the reduction of stroke risk and systemic embolism in nonvalvular atrial fibrillation, treatment of deep vein thrombosis (DVT) and pulmonary embolism (PE), and reduction in the risk of recurrence of DVT or PE [6]. Landmark trials such as ROCKET-AF were able to establish non-inferiority when compared to warfarin in the prevention of stroke or non-CNS systemic embolism [7]. Similar results were also seen in the treatment of DVT and PE when compared to enoxaparin or vitamin K antagonist (EINSTEIN PE and EINSTEIN DVT) [8,9]. In a meta-analysis by Liu et al. in 2023, it was even suggested that rivaroxaban significantly reduced the incidence of venous thromboembolism (VTE) compared to warfarin [10].

When prescribing rivaroxaban, the risk of these thrombotic events should be weighed against the increased risk of serious or fatal bleeding [6]. In the ROCKET-AF trial, it was demonstrated that the overall bleeding risk was not significantly different between rivaroxaban and warfarin. However, in this same study, rivaroxaban had an increased risk for gastrointestinal bleeding compared to warfarin (3.2% vs. 2.2% per year, p < 0.001), while the arguably more serious complications such as intracranial hemorrhage (0.5 vs. 0.7, p = 0.019) and fatal bleeding (0.2% vs. 0.5%, p = 0.003) were seen to be higher among patients in the warfarin group [10].

Spinal hematoma is a rare and often severe neurological disorder that can lead to death or permanent neurological deficit in the absence of adequate treatment, and literature offers no reliable estimates of its incidence, perhaps because of its rarity [11]. Spinal hematoma can be classified depending on location: epidural, intradural (subdural), subarachnoid, or intramedullary [11]. Particularly, SSDH represents approximately 4.1% of spinal hematoma cases [11]. Identified causes include coagulopathies, vascular malformations, trauma, procedures such as lumbar puncture and spine surgery [12], and even idiopathic in some cases [13].

Spontaneous spinal subdural hemorrhage associated with rivaroxaban use is rare, and there have only been a few documented cases [5,14,15]. These studies have observed different sites of involvement (cervicothoracic, thoracic, and thoracolumbar), as well as different approaches to treatment and outcomes. Zaarour et al. [5] described a patient maintained on rivaroxaban who developed nontraumatic subdural hemorrhage and was given high-dose steroids prior to undergoing surgery on the fourth hospital day. The patient showed gradual improvement, but recovery was incomplete. However, two other similar cases failed to show improvement after surgical intervention. One study made use of administering prothrombin complex concentrate followed by surgery the next day [14]. Meanwhile, in Castillo et al., cervical and lumbar drainage procedures were employed but showed no improvement in neurological functions after six months [15].

Owing to the rarity of the disease, the pathophysiology of spontaneous SSDH is still poorly understood. This becomes even more puzzling as the subdural cavity of the spinal cord lacks any bridging veins. Associated factors include coagulation dyscrasias, spinal procedures, and the use of anticoagulants [3]. However, anticoagulant therapy alone is unlikely to trigger spinal hemorrhage, and it is possible that there must be a region of decreased resistance together with increased pressure in the interior vertebral venous plexus to cause spinal hemorrhage [11]. One possible mechanism could be the sudden increase in pressure in the thoracic and/or abdominal cavities that leads to the rupture of these spinal vessels [16]. In our particular case, the use of anticoagulation, coupled with prolonged standing, which significantly increases intra-abdominal pressure [17], may have led to spontaneous SSDH. This combination was similarly postulated in the case described by Zaarour et al., but increased intra-abdominal pressure was thought to be because of seatbelt use for more than six hours during driving [5]. In our patient, the absence of any history of trauma, blood dyscrasias, use of epidural catheters, and/or spinal procedures exclude these factors’ possible contributory role in the pathology of the patient’s disease.

The treatment of SSDH associated with anticoagulant use remains unclear. Currently, there is no consensus or evidence-based guidelines for its treatment. Immediate discontinuation of the anticoagulant should be considered, especially if there are plans for surgical intervention. The role of reversal agents or antidotes should also be considered. Particularly for rivaroxaban, andexanet alfa is an FDA-approved reversal agent [18]. Prothrombin complex concentrate may also be an option as it may completely reverse the anticoagulant effect of rivaroxaban [19]. In a systematic review done on traumatic SSDHs, surgical evacuation was done on 32 patients with the most common indications including the presence of focal neurological deficits (46.9%), worsening neurological exam (31.3%), and failure of conservative management (6.3%) [20]. In this study, it was seen that surgical intervention was associated with poorer outcomes among those who presented with symptoms of weakness and neurological deficits on examination and were more likely to have evidence of direct spine trauma and spinal cord compression [20]. Meanwhile, conservative management seemed to suffice in the 26 other patients reviewed in the study [20]. Particularly for spontaneous spinal subdural hemorrhages, there have been mixed outcomes following surgical intervention [5,14,15]. The lack of any strong recommendations both for the medical and surgical management of spontaneous SSDH should warrant further investigation in the future.

## Conclusions

There has been a growing popularity for the use of oral anticoagulants in place of warfarin. Particularly for rivaroxaban, this is partly because of present clinical trials demonstrating a lower incidence of major bleeding events when compared with warfarin. However, spontaneous SSDH is a rare neurological emergency that may occur with its use. Patients maintained on rivaroxaban who present with acute onset severe back pain and neurological impairment should immediately be suspected of spontaneous SSDH as it could lead to permanent neurological impairment. Hence, establishing a proper and evidence-based approach to its treatment is imperative at this time.
